# Risk Factors of *Candida parapsilosis* Catheter-Related Bloodstream Infection

**DOI:** 10.3389/fpubh.2021.631865

**Published:** 2021-08-12

**Authors:** Dina Hussein Yamin, Azlan Husin, Azian Harun

**Affiliations:** ^1^Department of Medical Microbiology and Parasitology, School of Medical Sciences, Universiti Sains Malaysia, Kubang Kerian, Malaysia; ^2^Department of Medicine, School of Medical Sciences, Universiti Sains Malaysia, Kubang Kerian, Malaysia; ^3^Hospital Universiti Sains Malaysia, Kota Bharu, Malaysia

**Keywords:** *candida parapsilosis*, candidemia, catheter-related bloodstream infection, Malaysia, risk factors

## Abstract

Catheter-related bloodstream infection (CRBSI) is an important healthcare-associated infection caused by various nosocomial pathogens. *Candida parapsilosis* has emerged as a crucial causative agent for the CRBSI in the last two decades. Many factors have been associated with the development of CRBSI including, demography, pre-maturity, comorbidities (diabetes mellitus, hypertension, heart diseases, neuropathy, respiratory diseases, renal dysfunction, hematological and solid organ malignancies, and intestinal dysfunction), intensive care unit (ICU) admission, mechanical ventilation (MV), total parenteral nutrition (TPN), prior antibiotic and/or antifungal therapy, neutropenia, prior surgery, immunosuppressant, and type, site, number, and duration of catheters. This study aims to determine *C. parapsilosis* CRBSI risk factors. A retrospective study has been performed in an 853-bedded tertiary-care hospital in north-eastern Malaysia. All inpatients with *C. parapsilosis* positive blood cultures from January 2006 to December 2018 were included, and their medical records were reviewed using a standardized checklist. Out of 208 candidemia episodes, 177 had at least one catheter during admission, and 31 cases had not been catheterized and were excluded. Among the 177 cases, 30 CRBSI cases were compared to 147 non-CRBSI cases [81 bloodstream infections (BSIs), 66 catheter colonizers]. The significance of different risk factors was calculated using multivariate analysis. Multivariate analysis of potential risk factors shows that ICU admission was significantly associated with non-CRBSI as compared to CRBSI [OR, 0.242; 95% CI (0.080–0.734); *p* = 0.012], and TPN was significantly positively associated with CRBSI than non-CRBSI [OR, 3.079; 95%CI (1.125–8.429); *p* = 0.029], while other risk factors were not associated significantly. Patients admitted in ICU were less likely to develop *C. parapsilosis* CRBSI while patients receiving TPN were more likely to have *C. parapsilosis* CRBSI when compared to the non-CRBSI group.

## Introduction

*Candida* species have been reported as one of the most common opportunistic pathogens and as the fourth most common causes of bloodstream infections (BSIs) among patients who were hospitalized immunocompromised worldwide in the last two decades (1–4). In the USA, *Candida* species account for 8–15% of the total nosocomial BSIs. Invasive candidiasis is a global problem, with increasing incidence in Europe, Asia, Australia, and Latin America (4). Candidemia is a life-threatening infection associated with high crude and attributable mortality rates along with increased healthcare costs and prolonged duration of hospitalization (2, 3).

Recently, the implementation of invasive monitoring and treatment techniques has been escalated in the intensive care units (ICUs), and this eventually elevated the ratio of patients prone to fungal infections (5). Many risk factors associated with the development of invasive candidiasis, mainly candidemia, have been identified: broad-spectrum antibiotics, presence or prolonged use (6) of central venous catheters (CVCs) and other invasive devices (3), mechanical ventilation (MV), administration of total parenteral nutrition (TPN), hematological and solid organ malignancies, neutropenia, and acute renal failure (ARF) (1), prior fungal colonization (7), immunosuppressive therapies (e.g., chemotherapy, corticosteroids), admission to ICU and complicated surgery (6). Assessing these risk factors is integral for the early administration of proper antifungal therapeutics and hence reducing the burden of infection (1).

Many studies have reported an important shift in the types of *Candida* infection away from *Candida albicans* to more treatment-resistant, non-*albicans Candida* species (1). The emergence of non-*albicans Candida* species has been reported to cause a dramatic change in the spectrum of candidemia.

*Candida parapsilosis* is often the second most common *Candida* species isolated from the blood cultures (4). *Candida parapsilosis* BSIs are associated with indwelling catheters and TPN due to their capability to form biofilm on the surfaces of intravascular devices and on the hands of healthcare workers (4).

Long-term CVC placement has become widely available, such as implanted Port-A-Cath catheters and subcutaneously tunneled catheters. They enable direct and easy venous access for drug delivery, parenteral nutrition administration, blood transfusion, and laboratory testing for a long period of time (8). The use of catheters, especially CVCs, among patients in the ICU is common, and these catheters facilitate the entrance of pathogens, such as *Candida* species, causing candidemia. CVC placement can significantly increase the risk of candidemia in hospitalized patients and is an independent risk factor for candidemia (9). BSI is the most common life-threatening complication resulting from the central venous access either by colonizing the implanted catheters or by contaminating the catheter hub or infusions passing through those devices (8, 10). However, the advantages of using catheters outweigh their complications and their advantages are indisputable. CVCs are healthcare devices that are of great importance and that play an essential role in the management of patients who are critically ill, especially those in the ICU (11). The use of CVCs is an essential feature of the newly introduced medicine in patients who are critically ill. They allow hemodynamic monitoring and provide venous access for fluids, therapies, TPN, and blood products administration (12, 13) and access for blood specimens (10).

Even though, the incidence of catheter-related bloodstream infections (CRBSIs) is rising annually. CRBSI significantly increases the length of stay and hospital costs, which has an impact on the prognosis and quality of life of the patients, besides increasing the mortality rates (13). In Europe and America, many risk factors, in addition to the use of CVCs as well as having some underlying diseases specifically, have been identified to be associated with CRBSI: cancer, surgical trauma, and diabetes mellitus, receiving parenteral nutrition, vancomycin use, applying urinary catheters, age, and acute physiology and chronic health evaluation (APACHE) score. *Candida parapsilosis* accounts for 15–20% of the candidemia worldwide and is frequently associated with CRBSI (9).

Procedures for the prevention of CRBSI can be more efficient if we are aware of the potential risk factors (10). Currently, there are a limited number of studies on *C. parapsilosis* CRBSI. Most of the studies conducted to date have widely evaluated invasive candidiasis, while few studies emphasized on the CRBSI caused by *C. parapsilosis* specifically.

This retrospective study was performed to describe the epidemiology and the demographic and clinical characteristics of patients with candidemia caused by *C. parapsilosis* and to evaluate the risk factors of CRBSI due to *C. parapsilosis* in patients admitted to a tertiary teaching hospital in Malaysia from 2006 to 2018.

## Materials and Methods

### Study Design

This is a cross-sectional retrospective study conducted at Hospital Universiti Sains Malaysia (USM) from January 1, 2006 to December 31, 2018 to describe the clinical characteristics of the inpatients with *C. parapsilosis* candidemia and to determine the risk factors associated with CRBSI due to *C. parapsilosis* during the period of study. This study was approved by the Human Research Ethics of Universiti Sains Malaysia (JEPeM-USM-16040162).

### Inclusion and Exclusion Criteria

All patients, with at least one *C. parapsilosis* positive blood culture, admitted to Hospital USM during the study period were included. Any episode that occurred after at least 30 days from the first episode was considered as a new episode. Patients who had second candidemia episodes within the 30 days period were excluded. In the determination of *C. parapsilosis* CRBSI risk factors, patients who were not catheterized during their admission were excluded.

### Clinical and Laboratory Data

Data regarding *C. parapsilosis* isolation from blood samples and clinical information of the patients were obtained from the Hospital USM Laboratory Information System and Medical Records Unit using a standardized checklist. Data regarding demography of the patients (age, sex, and race), underlying diseases and comorbidities (pre-maturity, diabetes mellitus, hypertension, heart diseases, neuropathy, hematological and solid organ malignancies, respiratory diseases, renal dysfunction, intestinal diseases, hepatitis, abscesses, and burns), pre-disposing factors such as prior broad-spectrum antibiotic administration, central venous catheterization, data of vascular access [peripheral or central, insertion site, type of catheter(s) used, number of replaced CVCs, and duration], ICU admission, MV, TPN, neutropenia, prior surgeries, receipt of immunosuppressive drugs (chemotherapy, corticosteroids), antifungal prophylaxis, antifungal treatment, laboratory tests [C-reactive protein level (CRP), total white blood cell count (TWBC), absolute neutrophil count (ANC), platelet count] that were done on the same date of diagnosis, and outcomes (microbiological cure or death) were recorded from the date of admission until the index day (the hospital day on which the first sample positive for *C. parapsilosis* by culture was collected).

### Isolation and Species Identification

The isolation, culture, and species identification of *C. parapsilosis* were performed using standard methods in the mycology laboratory of Hospital USM. Blood was drawn simultaneously from the catheter and a peripheral vein, and separate blood culture bottles were inoculated with an equal amount of each blood sample. The incubation of the blood cultures took place in an automated system (BACTEC 9000 system™, Becton Dickinson, USA) and positive blood bottles were subcultured on Sabouraud Dextrose Agar (SDA, Oxoid, UK) plates. Commercially available identification systems, namely, API 20C AUX® or ID 32C® biochemical identification kit (BioMérieux, Marcy I'Etoile, France) or VITEK® system (BioMérieux, Marcy I'Etoile, France) (last 2 years), have been used for the identification of *Candida* to species level. Molecular identification by the internal-transcribed spacer (ITS) region of ribosomal DNA sequencing was also performed in selected cases.

### Statistical Analysis

Data were analyzed using SPSS for Windows (Version 24.0; SPSS, Chicago, IL). Categorical variables were presented as proportions and percentages, while numerical variables were presented as mean, SD or median, and interquartile ranges according to its distribution. Univariate data analysis was done to compare CRBSI risk factors among two groups (non-CRBSI and CRBSI) using the chi-square test or the Fisher exact-test for categorical variables as appropriate and an independent *t*-test or the Mann–Whitney *U*-test for numerical variables as appropriate. A multivariate analysis was done to determine the independent risk factors of CRBSI, where all risk factors with *p* < 0.25 from an univariate analysis and other clinically important factors were included in the multiple logistic regression model. All the variables were two-tailed. A value of *p* < 0.05 was considered to be statistically significant.

### Definitions

An episode of candidemia caused by *C. parapsilosis* was identified when the *C. parapsilosis* was isolated from at least one blood culture (peripheral or central) of the patient. Episodes of candidemia in a single patient were considered distinct if they occurred at least 30 days apart. *Candida parapsilosis* BSI definition requires isolation of *C. parapsilosis* from at least one peripheral blood sample with or without positive central blood (14). According to the CDC and IDSA guidelines (15, 16), CRBSI was defined if the same microorganism was isolated from a venipuncture drawn blood culture and from a blood culture catheter drawn at least 2 h earlier. CRBSI is defined precisely as “infection due to a central venous catheter.” It includes infection due to non-tunneled CVCs, tunneled CVCs, Hickman catheters, peripherally inserted central catheters (PICC) or port-a-cath (port), triple lumen catheters, or umbilical venous catheter. It excludes infections due to arterial catheters, peripheral venous catheters, or urinary catheters (15). Neutropenia was defined as ANC <500 cells/mL. Long antibiotic administration was considered if the patient received antibiotics for > 14 days during the month prior to the occurrence of the candidemia episode, while short antibiotic administration was considered if the patient received antibiotics for 1–14 days during that month. Broad-spectrum antibiotics include fourth-generation cephalosporins and carbapenems. Microbiological recovery was defined as negative blood cultures for at least 2 consecutive days without any subsequent positive blood cultures. As complete data on catheter tip culture were not available, isolation of *C. parapsilosis* from central blood sample alone is considered as catheter colonizer.

## Results

A total of 208 episodes of candidemia caused by *C. parapsilosis* were identified among 197 patients from January 2006 to December 2018 at Hospital USM, Malaysia. One hundred ninety patients had one episode and seven patients had more than one episode (one patient had four episodes, two patients had three episodes, and four patients had two episodes). Of the 208 episodes, 112 (53.8%) were BSIs, 66 (31.8%) were catheter colonizers, and only 30 (14.4%) were CRBSIs.

The demographics, clinical characteristics, and pre-disposing factors of the 208 episodes of *C. parapsilosis* candidemia are summarized in Table 1. One hundred twenty (57.7%) patients were men, and 88 (42.3%) were women. Thirty six patients (17.3%) were younger than 1 year old, 73 (35.1%) were 1–19 years old, and 36 (17.3%) were 60 years of age or older. Most of the patients were Malaysians. Most of the patients had at least one underlying disease. The most common underlying diseases were malignancy (*n* = 93, 44.7%), including hematological malignancies (*n* = 51, 24.5%) and solid organ tumors (*n* = 43, 20.7%), hypertension (*n* = 52, 25.0%), renal dysfunctions (*n* = 48, 23.1%), and diabetes mellitus (*n* = 43, 20.7%). Other underlying diseases, such as gastrointestinal, cardiovascular, neurological, and pulmonary diseases, were less encountered. Hepatitis, abscesses, burns, and pre-maturity were rarely encountered. Out of 208 episodes, prior antibiotics were received by 143 (68.7%) patients, out of whom 35 (16.8%) received at least one antibiotic for more than 14 days and 108 (51.9%) received antibiotics for 14 days or less. One hundred seventy-seven (85.1%) patients were catheterized at least one time, out of whom 86 (41.3%) were catheterized for more than 30 days, most of them used one type (or site) of catheter [139 (66.8%)], 30 (14.5%) patients were catheterized by two different types (or sites) of catheter, only 8 (3.8%) used three types. Chemoport, internal jugular, and femoral catheters were used almost equally in 64 (30.8%), 64 (30.8%), and 62 (29.8%) patients, respectively. PICC, subclavian, and umbilical catheters were used rarely. Eighty-eight (42.3%) patients were admitted to ICU, 70 (33.7%) underwent MV, 55 (26.4%) received TPN, 59 (28.5%) had neutropenia, 73 (35.1%) had prior surgery, and 120 (57.7%) were treated with immunosuppressive drugs. Death was the outcome of 41 (19.7%) patients, while 167 (80.3%) patients were cured microbiologically.

**Table 1:** The demographics, clinical characteristics, and predisposing factors of 208 patients with *Candida parapsilosis* candidemia.

**Variable**	**Frequency (%)**
**Gender**	
Male	120 (57.7)
Female	88 (42.3)
**Age ranges**	
Below 1	36 (17.3)
1–19	73 (35.1)
20–39	30 (14.4)
40–59	33 (15.9)
60 and above	36 (17.3)
**Ethnicity**	
Malay	198 (95.2)
Chinese	8 (3.8)
Others	2 (1.0)
**Underlying diseases:**	
Pre-mature	6 (2.9)
Diabetes mellitus	43 (20.7)
Hypertension	52 (25.0)
Cardiovascular diseases^a^	23 (11.1)
Neurological diseases^b^	22 (10.6)
Malignancy	93 (44.7)
Solid organ tumor	43 (20.7)
Hematological malignancy	51 (24.5)
Chronic pulmonary diseases^c^	11 (5.3)
Renal dysfunction^d^	48 (23.1)
Gastrointestinal diseases^e^	29 (13.9)
Abscess or burns	8 (3.8)
Hepatitis	10 (4.8)
**Antibiotic administration**	
No prior antibiotics given	65 (31.3)
14 days or less	108 (51.9)
More than 14 days	35 (16.8)
**Number of antibiotic given > 14 days**	
No	173 (83.2)
1	24 (11.5)
2	8 (3.8)
3	2 (1.0)
4	1 (0.5)
Catheterization	177 (85.1)
**Number of catheter/types**	
0	31 (14.9)
1	139 (66.8)
2	30 (14.5)
3	8 (3.8)
Internal jugular	64 (30.8)
Subclavian	8 (3.8)
Femoral	62 (29.8)
Umbilical venous	8 (3.8)
Chemoport	64 (30.8)
PICC	9 (4.3)
CVL	9 (4.3)
Catheterization > 30 days	86 (41.3)
ICU admission	88 (42.3)
Mechanical ventilation	70 (33.7)
Total parenteral nutrition	55 (26.4)
Neutropenia^f^	59 (28.5)
Total white blood cell, mean (SD)	8.28 (8.33)
Neutrophils count, mean (SD)	3.90 (6.92)
Platelet count, mean (SD)	189 (200)
Prior surgery	73 (35.1)
Immunosuppressive therapy^g^	120 (57.7)
**Outcome**	
Death	41 (19.7)
Microbiological cure	167 (80.3)
**Microbiological diagnosis**	
BSI	112 (53.8)
Catheter colonizer	66 (31.8)
CRBSI	30 (14.4)

a *Cardiovascular diseases include heart failure, ischemic heart disease, endocarditis, and arrhythmia*.

b *Neurological diseases include Parkinson's disease, Alzheimer's disease, epilepsy, and paralysis*.

c *Chronic pulmonary diseases include asthma, chronic bronchitis, emphysema, and lung fibrosis*.

d *Renal Dysfunctions include end-stage renal failure (ESRF) and chronic kidney disease*.

e *Gastrointestinal diseases include Crohn's disease, ulcerative colitis, chronic pancreatitis, and gallbladder stones*.

f *Neutropenia is defined as absolute neutrophil count (ANC) <500 cell/mL*.

g *Immunosuppressive therapy includes corticosteroids and chemotherapy*.

Among the 177 episodes that were identified in patients who were catheterized, after excluding 31 episodes that occurred in patients who were non-catheterized, there were 30 episodes of CRBSI and 147 episodes of non-CRBSI (composed of 81 BSI and 66 catheter colonizer), as shown in Table 2. All the potential risk factors (demographic, underlying diseases, and invasive procedures) were included in a univariate analysis for comparison between patients with CRBSI and non-CRBSI. There was a significant difference in the number of catheter types used between patients with CRBSI and non-CRBSI (*p* = 0.036), most patients with CRBSI 26 (86.7%) used catheter(s) of one type, and only four patients with CRBSI used two or three different types of catheters, while 113 (76.9%) of the patient with non-CRBSI used catheter(s) of one type, and 34 (23.1%) used two or three different catheter types. There was a significant difference in ICU admission between CRBSI and non-CRBSI (*p* = 0.025), higher percentage of patients who developed non-CRBSI [72 (49.0%)] were admitted to ICU when compared with 8 CRBSIs (26.7%). Patients with malignancies developed CRBSI more often than non-CRBSI, 19 (63.3%) vs. 67 (45.6%), respectively, but the difference was not significant (*p* = 0.076). The duration of catheterization differed between patients with CRBSI and non-CRBSI, 18 (60.0%) vs. 68 patients (46.3%), respectively, were catheterized > 30 days, but the difference was also not significant (*p* = 0.170). Patients with non-CRBSI had MV more than the patients with CRBSI, 59 (40.1%) vs. 7 (23.3%), respectively, but the difference was not significant (*p* = 0.083). There was no significant difference between CRBSI and non-CRBSI in the term of receiving TPN (*p* = 0.187). Other potential risk factors were analyzed but were not significant in the univariate analysis including gender, age, ethnicity, pre-maturity, diabetes mellitus, hypertension, cardiovascular, neurological, pulmonary, renal, gastrointestinal diseases, abscesses, burns, hepatitis, prior antimicrobial (antibiotics and antifungal) treatment, neutropenia, prior surgery, and immunosuppressive therapy. Four laboratory test values were included in the univariate analysis. CRBSI group had lower TWBC as compared with non-CRBSI, but the difference was not significant (*p* = 0.068). No significant difference in CRP, ANC, and platelet count was noted between the two groups. Death was the outcome of 6 (20.0%) and 32 (21.8%) patients with CRBSI and non-CRBSI, respectively, but the difference was not significant (Table 2).

**Table 2:** Comparison between patients with catheter-related bloodstream infection (CRBSI) and those with non-CRBSI caused by *C. parapsilosis*.

**Variable**	**Total**	**CRBSI**	**non-CRBSI**	**Test Statistics**	***p*-value**
	***n* = 177**	***n* (%)**	***n* (%)**		
		***N* = 30**	***N* = 147**		
**Gender**				0.735 (1)	0.391^a^
Male	101	15 (50.0)	86 (58.5)		
Female	76	15 (50.0)	61 (41.5)		
**Age ranges**					0.396^b^
Below 1	27	4 (13.3)	23 (15.6)		
1–19	65	15 (50.0)	50 (34.1)		
20–39	27	4 (13.3)	23 (15.6)		
40–59	30	2 (6.7)	28 (19.1)		
60 and above	28	5 (16.7)	23 (15.6)		
**Ethnicity**					0.266^b^
Malay	168	29 (96.7)	139 (94.5)		
Chinese	7	0 (0.0)	7 (4.8)		
Others	2	1 (3.3)	1 (0.7)		
**C-Reactive protein**	144			0.258 (1)	0.611^a^
Negative	35	6 (20.7)	29 (25.2)		
Positive	109	23 (79.3)	86 (74.8)		
**Underlying disease:**					
Pre-mature	6	0 (0.0)	6 (4.1)		0.591^b^
Diabetes mellitus	38	7 (23.3)	31 (21.1)	0.074 (1)	0.785^a^
Hypertension	46	5 (16.7)	41 (27.9)	1.632 (1)	0.201^a^
Cardiovascular diseases	19	3 (10.0)	16 (10.9)		>0.950^b^
Neurological diseases	17	2 (6.7)	15 (10.2)		0.741^b^
Malignancy	86	19 (63.3)	67 (45.6)	3.144 (1)	0.076^a^
Solid organ tumor	40	9 (30.0)	31 (21.1)	1.131 (1)	0.288^a^
Hematological malignancy	47	10 (33.3)	37 (25.2)	0.851 (1)	0.356^a^
Chronic pulmonary diseases	10	1 (3.3)	9 (6.1)		>0.950^b^
Renal dysfunction	43	5 (16.7)	38 (25.9)	1.143 (1)	0.285^a^
Gastrointestinal diseases	27	7 (23.3)	20 (13.6)		0.175^b^
Abscess or burns	7	1 (3.3)	6 (4.1)		>0.950^b^
Hepatitis	9	1 (3.3)	8 (5.4)		>0.950^b^
**Antibiotic administration**				1.389 (2)	0.499^a^
No prior antibiotics given	44	10 (33.3)	34 (23.1)		
14 days or less	100	15 (50.0)	85 (57.9)		
More than 14 days	33	5 (16.7)	28 (19.0)		
**Number of antibiotics given >14 days**	33				0.119^b^
1	24	2 (40.0)	22 (78.6)		
2	7	3 (60.0)	4 (14.2)		
3	1	0 (0.0)	1 (3.6)		
4	1	0 (0.0)	1 (3.6)		
Duration for long antibiotic	33			43.500	0.180^c^
Duration of antifungal treatment before isolation	34			61.000	0.296^c^
**Number of catheter types**				6.656 (2)	0.036^a^
1	139	26 (86.7)	113 (76.9)		
2	30	1 (3.3)	29 (19.7)		
3	8	3 (10.0)	5 (3.4)		
**Type of catheters used:**					
Internal jugular	64	10 (33.3)	54 (36.7)	0.125 (1)	0.724^a^
Subclavian	8	1 (3.3)	7 (4.8)		>0.950^b^
Femoral	62	8 (26.7)	54 (36.7)	1.110 (1)	0.292^a^
Umbilical venous	8	0 (0.0)	8 (5.4)		0.355^b^
Chemoport	64	15 (50.0)	49 (33.3)	2.998 (1)	0.083^a^
PICC	9	3 (10.0)	6 (4.1)		0.180^b^
CVL	9	0 (0.0)	9 (6.1)		0.360^b^
Catheterization > 30 days	86	18 (60.0)	68 (46.3)	1.883 (1)	0.170^a^
ICU admission	80	8 (26.7)	72 (49.0)	5.008 (1)	0.025^a^
Mechanical ventilation	66	7 (23.3)	59 (40.1)	3.008 (1)	0.083^a^
Total parenteral nutrition	53	12 (40.0)	41 (27.9)	1.741 (1)	0.187^a^
Neutropenia	56	12 (40.0)	44 (30.1)	1.116 (1)	0.291^a^
Total white blood cell, mean (SD)	176	6.75 (5.23)	9.77 (8.69)	1.838	0.068^d^
Neutrophils count, mean (SD)	87	4.55 (5.03)	4.85 (5.58)	0.216	0.829^d^
Platelet count, mean (SD)	176	221.70 (179.02)	207.36 (161.32)	−0.435	0.664^d^
Prior surgery	64	13 (43.3)	51 (34.7)	0.806 (1)	0.369^a^
Immunosuppressive therapy	114	21 (70.0)	93 (63.3)	0.493 (1)	0.483^a^
**Outcome**				0.046 (1)	0.830^a^
Death	38	6 (20.0)	32 (21.8)		
Microbiological cure	139	24 (80.0)	115 (78.2)		

a *Chi-square value*.

b *Fisher Exact-test*.

c *Mann–Whitney U-test*.

d *Independent t-test*.

A simple logistic regression analysis of the 177 episodes of *C. parapsilosis* CRBSI and non-CRBSI (Table 3) shows that hypertension, malignancy, gastrointestinal diseases, duration of antifungal treatment before isolation, number of catheter types, use of chemoport, use of PICC, catheterization > 30 days, ICU admission, MV, TPN receipt, and TWBC had *p* < 0.250 and, therefore, were included in the multivariate analysis in addition to the clinically important factors such as prior broad-spectrum antibiotic administration. Based on this simple logistic regression analysis, there was a significant association between giving two different antibiotics for more than 14 days and the development of CRBSI [OR, 8.250 at 95% CI (1.028–66.192), *p* = 0.047]. ICU admission was significantly negatively associated with the development of CRBSI. Patients admitted to ICU were more likely to have non-CRBSI than CRBSI [OR, 0.379, 95% CI (0.158–0.905), *p* = 0.029]. Other factors were not statistically significant.

**Table 3:** Univariate analysis of *C. parapsilosis* CRBSI risk factors.

**Variable**	**Coefficient of regression, B**	**Crude OR (95% CI)**	***p*-value**
**Gender**			
Male	0	1.000	
Female	0.343	1.410 (0.642–3.098)	0.393
**Age ranges**			
Below 1	0	1.000	0.427
1–19	0.545	1.725 (0.515–5.776)	0.377
20–39	0	1.000 (0.223–4.489)	>0.950
40–59	−0.890	0.411 (0.069–2.447)	0.328
60 and above	0.223	1.250 (0.297–5.256)	0.761
**Ethnicity**			
Malay	0	1.000	0.548
Chinese	−19.636	0.000	0.999
Others	1.567	4.793 (0.291–78.863)	0.273
C-reactive protein (Y/N)	0.257	1.293 (0.479–3.486)	0.612
Underlying disease^*^			
Pre-mature	−19.655	0.000	0.999
Diabetes mellitus	0.130	1.139 (0.447–2.899)	0.785
Hypertension	−0.660	0.517 (0.185–1.442)	0.208
Cardiovascular diseases	−0.095	0.910 (0.248–3.341)	0.887
Neurological diseases	−0.464	0.629 (0.136–2.905)	0.552
Malignancy	0.724	2.062 (0.917–4.638)	0.080
Solid organ tumor	0.472	1.604 (0.668–3.850)	0.290
Hematological Malignancy	0.396	1.486 (0.638–3.462)	0.358
Chronic pulmonary diseases	−0.637	0.529 (0.064–4.337)	0.553
Renal dysfunction	−0.556	0.574 (0.205–1.605)	0.290
Gastrointestinal diseases	0.659	1.933 (0.734–5.091)	0.182
Abscess or burns	−0.210	0.810 (0.094–6.987)	0.848
Hepatitis	−0.512	0.599 (0.072–4.977)	0.635
**Antibiotic administration**			
No prior antibiotics given	0	1.000	0.504
14 days or less	−0.511	0.600 (0.246–1.466)	0.263
More than 14 days	−0.499	0.607 (0.186–1.984)	0.409
**Number of antibiotics given >14 days**			
1	0	1.000	0.267
2	2.100	8.250 (1.028–66.192)	0.047
3	−18.805	0.000	>0.950
4	−18.805	0.000	>0.950
Duration for long antibiotic	0.061	1.063 (0.964–1.172)	0.219
Duration of antifungal treatment before isolation (17)	0.019	1.020 (0.959–1.084)	0.536
**Number of catheter types**			
1	0	1.000	0.074
2	−1.898	0.150 (0.020–1.151)	0.068
3	0.958	2.608 (0.586–11.611)	0.208
Type of catheters used^*^			
Internal jugular	−0.150	0.861 (0.376–1.975)	0.724
Subclavian	−0.372	0.690 (0.082–5.821)	0.733
Femoral	−0.468	0.626 (0.261–1.504)	0.295
Umbilical venous	−19.670	0.000	0.999
Chemoport	0.693	2.000 (0.904–4.423)	0.087
PICC	0.960	2.611 (0.615–11.084)	0.193
CVL	−19.677	0.000	0.999
Catheterization > 30 days^*^	0.555	1.743 (0.784–3.875)	0.173
ICU admission^*^	−0.971	0.379 (0.158–0.905)	0.029
Mechanical ventilation^*^	−0.790	0.454 (0.183–1.126)	0.088
Total parenteral nutrition^*^	0.544	1.724 (0.763–3.892)	0.190
Neutropenia^*^	0.435	1.545 (0.686–3.479)	0.293
Total white blood cell	−0.064	0.938 (0.877–1.004)	0.067
Neutrophils count	−0.011	0.989 (0.898–1.090)	0.827
Platelet count	0.001	1.001 (0.998–1.003)	0.662
Prior surgery^*^	0.364	1.439 (0.648–3.197)	0.371
Immunosuppressive therapy^*^	0.304	1.355 (0.579–3.169)	0.484
**Outcome**			
Death	0	1.000	
Microbiological cure	0.107	1.113 (0.419–2.956)	0.830

* *Simple logistic regression reference groups are patients without these variables*.

*Df, degree of freedom; OR, Odd Ratio; CI, Confidence Interval*.

In a multivariate analysis for the risk factors of *C. parapsilosis* CRBSI (Table 4), admission to ICU and receipt of TPN were significant independent risk factors for *C. parapsilosis* CRBSI. Patients with CRSBI were less likely to be admitted to ICU [adjusted OR, 0.192, 95% CI (0.064–0.551), *p* = 0.002] when compared to patients with non-CRBSI. However, patients with CRBSI were more likely to be given TPN [adjusted OR, 3.605, 95% CI (1.353–9.606), *p* = 0.010] compared to patients with non-CRBSI.

**Table 4:** Multivariate analysis of *C. parapsilosis* CRBSI risk factors.

**Risk factors**	**Coefficient of regression, B**	**Wald (df)**	**Adjusted OR (95% CI)**	***p*-value**
ICU Admission	−1.651	9.412 (1)	0.192 (0.064–0.551)	0.002
Total parenteral nutrition	1.282	6.577 (1)	3.605 (1.353–9.606)	0.010

*df, degree of freedom; OR, Odd Ratio; CI, Confidence Interval*.

## Discussion

*Candida parapsilosis* is an emerging cause of candidemia in the last decades where the incidence of candidemia due to non-*albicans Candida* has significantly increased. Widespread use of invasive procedures, especially CVC, was the most common risk factor for *C. parapsilosis* candidemia because of the ability of this species to form biofilm on prosthetic materials such as CVC and of the ability to grow in a solution with high glucose concentration such as TPN fluids. In addition, *C. parapsilosis* is part of the normal flora that can be found on the skin of healthcare providers and hence can be transmitted to patients and cause infection in immunocompromised patients (9, 18, 19). *Candida parapsilosis* has been particularly implicated in the intravascular catheter-related infections in neonatal and pediatric age groups (20). There have been previously reported studies on the risk factors of CRBSI in general and *Candida*-related CRBSI (8–13, 21–27). Nevertheless, data on the risk factors of *C. parapsilosis* CRBSI is still scarce.

In this study, 208 episodes of *C. parapsilosis* candidemia were found in 197 patients admitted to Hospital USM, Malaysia, from which 14.4% were CRBSI. Strict adherence to CDC and IDSA definitions of CRBSI could be the cause of the relatively low percentage of CRBSI in this study, particularly, when it heavily relied on the documentation of time to positivity of central and peripheral samples. Nevertheless, the low-CRBSI percentage may have been a genuine phenomenon as a results of early removal and adequate catheter care. The most common underlying diseases were malignancies, either hematological or solid organ tumors, hypertension, renal dysfunctions, and diabetes mellitus. Others include prior antibiotics administration, admission to ICU, MV, receipt of TPN, neutropenia, prior surgery, and immunosuppressive therapy. Death was the outcome of 41 patients, while microbiological cure was the outcome in 167 patients. In a univariate analysis, there was a significant difference in the number of catheter types used between patients with CRBSI and those with non-CRBSI; in particular, more different types of catheters were used in patients with non-CRBSI as compared to patients with CRBSI. In addition, a significantly higher percentage of patients with non-CRBSI was admitted to ICU when compared to those with CRBSI. Malignancies were seen in patients with CRBSI more often than those with non-CRBSI, but the difference was not significant. A higher percentage of patients with CRBSI were catheterized for more than 30 days as compared to patients with non-CRBSI; however, the difference was not significant. In multivariate analysis, admission to ICU and receipt of TPN were significant independent risk factors for *C. parapsilosis* CRBSI. Patients with CRBSI were less likely to be admitted to ICU when compared to patients with non-CRBSI, whereas patients with CRBSI were more likely to be treated with TPN as compared to patients with non-CRBSI.

In our study, 177 (85.1%) out of 208 patients with *C.!parapsilosis* candidemia were catheterized at least one time. Similar to our results, Liu et al. reported that venous catheterization was present in over 90% of the patients, clearly indicating that venous catheterization was one of the major risk factors for *C. parapsilosis* candidemia (19). Also, in a study conducted in 16 pediatric patients with the ICU, candidemia was associated with CVC use, with specific *C. albicans, C. parapsilosis, C. tropicalis, C. krusei*, and *C. glabrata* identified in two, six, three, two, and three cases, respectively. CVC-related candidemia was also associated with the presence of CVC for more than 15 days, the use of an endotracheal tube (ETT) and MV, age over 1 year, and receipt of TPN (28). It has been shown that high percentages of patients with parenteral nutrition and central vascular access, especially in *C. parapsilosis* candidemia, were mostly isolated in internal medicine wards (IMW) where extensive use of intravascular devices was widely practiced (2).

In accordance with our results, several studies have demonstrated that TPN is a significant independent risk factor for candidemia and was associated independently with an increased risk of candidemia (5, 7, 29, 30). *Candida parapsilosis* is known to form a biofilm layer composed of glycosylated serum after adherence to bioprosthetic apparatus such as catheters and can cause outbreaks (17, 27, 31–35), and this can be possibly related to impaired catheter care, increase in the parenteral nutrition usage, or incompetent infection control practices. Moreover, candidemia was shown to be associated with longer ICU stay, and most of the patients were admitted to ICU for more than 15 days in the candidemia group compared with the non-candidemia group. ICU admission, CVC implementation, and administration of antimicrobial drugs before the onset of candidemia contributed significantly to the development of candidemia (3).

Patients in ICU were at particular risk of acquiring healthcare-associated infections, because they were critically ill and relatively immunocompromised. Furthermore, they are usually exposed to multiple pre-disposing factors such as the presence of CVC, hemodialysis, and receiving broad-spectrum antibiotics (36). In contrast, our finding that ICU admission is an independent risk factor for CRBSI, whereas a higher percentage of patients with non-CRBSI were in ICU than patients with CRBSI at the time of diagnosis, can be justified by practicing good preventative procedures such as replacement of contaminated catheter as fast as possible to prevent further development of more serious catheter complications like CRBSI. Although candidemia is often associated with an ICU stay, only 88 patients (42.3%) were in the ICU when candidemia was diagnosed. In another study, only one-third of patients were in ICU, and this supports our finding that candidemia is not exclusively associated with critical care (18). Unlike our result, a multivariable analysis in another study revealed that only renal failure was an independent risk factor for CRBSI but not TPN (12). Another study observed that, in a multivariate analysis only, the use of multiple-lumen catheters and duration of CVC catheterization were found to be independent risk factors for CRBSI (11). Similarly, Ishizuka et al. concluded that femoral venous catheters are a major risk factor for patients with CRBSI undergoing colorectal surgery (37). In addition, secondary candidemia because of respiratory or urinary tract colonization, associated with MV or urine catheters, respectively, and intestinal translocation due to mucosal barrier injury could contribute to other sources of BSI.

One more author studied the risk factors of CRBSI (13). During that study, 21 patients had one CRBSI episode, 7 of them were fungemia, and only one was due *C. parapsilosis*. In multiple logistic regression, patients with multiple CVCs were nearly six times more likely to have CRBSI than patients with just one CVC. Those patients could be in fact more frequently sampled and transfused through these catheters as long as the catheter is present. This could increase the probability of introducing organisms through these catheters. In patients who received more than three different antibiotic regimens before diagnosis, CRBSI was seven times more likely to occur. Broad-spectrum antibiotic administration, especially quinolones and third-generation cephalosporin, led to inhibition of normal bacterial flora *in vivo* and alter flora equilibrium. Other factors such as type of catheter, site of catheter insertion, and TPN have no significant effect on the CRBSI incidence (13).

This study is a cross-sectional study in which the majority of the records are retrospective in nature. Though it has some limitations, it has provided important clinical epidemiological data regarding CRBSI caused by *C. parapsilosis*. A further large-scale prospective study on *C. parapsilosis* CRBSI is recommended to address all the potential risk factors, including the most common sources of colonization as well as follow-up healthcare. An understanding of CRBSI pathogenesis would help to direct efforts to create efficient prevention strategies.

## Conclusion

This is one of the few studies available globally in *C. parapsilosis* CRBSI that investigated risk factors, and data analysis demonstrated that hospitalization outside ICU and receipt of TPN were independent risk factors of *C. parapsilosis* CRBSI. In particular, patients admitted in the ICU were less likely to develop CRBSI, whereas patients receiving TPN were more likely to have CRBSI as compared to the non-CRBSI group.

## Data Availability Statement

The raw data supporting the conclusions of this article will be made available by the authors, without undue reservation.

## Ethics Statement

The studies involving human participants were reviewed and approved by Human Research Ethics Committee of Universiti Sains Malaysia (JEPeM-USM-16040162). Written informed consent from the participants' legal guardian/next of kin was not required to participate in this study in accordance with the national legislation and the institutional requirements.

## Author Contributions

DY conducted a review of records, carried out data analysis, and prepared the manuscript draft. AHu and AHa designed and supervised the study and revised the manuscript. All authors read and approved the final manuscript.

## Conflict of Interest

The authors declare that the research was conducted in the absence of any commercial or financial relationships that could be construed as a potential conflict of interest.

## Publisher's Note

All claims expressed in this article are solely those of the authors and do not necessarily represent those of their affiliated organizations, or those of the publisher, the editors and the reviewers. Any product that may be evaluated in this article, or claim that may be made by its manufacturer, is not guaranteed or endorsed by the publisher.
